# No evidence of conditioning of pupillary constriction despite overtraining

**DOI:** 10.7717/peerj.12948

**Published:** 2022-02-15

**Authors:** Diederick C. Niehorster, Stina Bengtsson, Niklas Brodin, Anders Rasmussen

**Affiliations:** 1 Lund University Humanities Lab, Lund University, Lund, Sweden; 2 Department of Psychology, Lund University, Lund, Sweden; 3 Department of Experimental Medical Science, Lund University, Lund, Sweden

**Keywords:** Classical conditioning, Pupillary constriction, Associative learning, Timing, Autonomic reflexes

## Abstract

Eyeblink conditioning is the most popular paradigm for studying classical conditioning in humans. But the fact that eyelids are under voluntary control means it is ultimately impossible to ascertain whether a blink response is ‘conditioned’ or a timed ‘voluntary’ blink response. In contrast, the pupillary response is an autonomic response, not under voluntary control. By conditioning the pupillary response, one might avoid potential volition-related confounds. Several attempts have been made to condition the pupillary constriction and dilation responses, with the earliest published attempts dating back to the beginning of the 20th century. While a few early studies reported successful conditioning of pupillary constriction, later studies have failed to replicate this. The apparatus for recording pupil size, the type of stimuli used and the interval between the stimuli has varied in previous attempts—which may explain the inconsistent results. Moreover, measuring the pupil size used to be cumbersome compared with today when an eyetracker can continuously measure pupil size non-invasively. Here we used an eyetracker to test whether it is possible to condition the autonomic pupillary constriction response by pairing a tone (CS) and a light (US) with a 1s CS-US interval. Unlike in previous studies, our subjects went through multiple training sessions to ensure that any potential lack of conditioning would not be due to too little training. A total of 10 participants went through 2–12 conditioning sessions, each lasting approximately 20 min. One training session consisted of 75 paired, tone + light, trials and 25 randomly interspersed CS alone trials. The eyetracker (Tobii Pro Nano), continuously measured participants’ pupil size. To test statistically whether conditioning of the pupillary response occurred we compared the pupil size after the tone on the first session and the last session. The results showed a complete lack of evidence of conditioning. Though the pupil size varied slightly between participants, the size did not change as a result of the training—irrespective of the number of training sessions. The data replicate previous findings that pupillary constriction does not show conditioning. We conclude that it is not possible to condition pupillary constriction—at least not by pairing a tone and a light. One hypothesis is that when pupillary conditioning has been observed in previous studies, it has been mediated by conditioning of an emotional response.

## Introduction

In classical conditioning, a neutral conditional stimulus (CS), for example a tone or a light, is repeatedly paired with an unconditional stimulus (US), that triggers a reflexive unconditional response (UR), for example salivation or an eyeblink. After a number of pairings, the CS will elicit a conditional response (CR)—even when it is not paired with the US. Ivan Pavlov was the first scientist to study classical conditioning systematically (Pavlov, 2010). Pavlov studied the dogs’ innate reflex of salivation, which was triggered by the smell of food (US). Pavlov observed that it was not only the smell of food that caused the dogs to salivate, but also the sound of the bell that Pavlov rang before the dogs were served their meal. Thus, the sound of the bell (CS) triggered the dogs’ autonomic salivatory response (CR). Since Pavlov’s seminal discoveries, classical conditioning has mainly focused on fear conditioning (LeDoux, 2000; Moore, 2002; Ernst et al., 2019) and eyeblink conditioning (McCormick & Thompson, 1984; Coleman & Webster, 1988; Moore, 2002; Löwgren, Bååth & Rasmussen, 2020). In humans, classical conditioning has mainly been studied using eyeblink conditioning. However, a recent study showed that timed blink responses can be produced voluntarily, without ever presenting a US (Rasmussen & Jirenhed, 2017). This highlights the yet unsolved problem of volition when studying eyeblink conditioning in humans (Kimble & Perlmuter, 1970; Coleman & Webster, 1988; Clark & Squire, 1998). That is, there is no way of knowing for sure if an adaptively timed blink response was produced voluntarily or if it was conditioned.

Unlike eyelid movements which can be initiated at will, humans do not normally have direct voluntary control of our pupils (Loewenfeld & Lowenstein, 1999). Thus, if it is possible to condition changes in pupillary size, this paradigm would offer a way to study classical conditioning without volitional contamination. However, previous research has shown that viewing stimuli associated with brightness, such as pictures of the sun, or imagining scenes of varying brightness, can affect pupil size (Binda, Pereverzeva & Murray, 2013; Laeng & Sulutvedt, 2014). According to these studies some neutral, non-visual stimuli can change the size of the pupil. Even more surprising, a recent paper describes a 23 year old student with the ability to voluntarily increase and decrease the size of his pupils (Eberhardt et al., 2021) despite the fact that direct control of pupillary musculature has previously been deemed impossible (Loewenfeld & Lowenstein, 1999). If changes in pupil size can be triggered by imagining scenes and, in rare cases, voluntarily, it is conceivable that changes in pupil size can be conditioned. If one could condition the size of the pupil, it would provide a simple associative learning paradigm less sensitive to volitional interference. Previous experiments have however yielded mixed results.

The first attempt to condition pupillary constriction was made by Cason (1922). He presented a light and as the pupil contracted, he presented a tone. Later, when he presented only the tone, he also observed a reduction in pupil size. This, Cason argued, showed that it is possible to condition reflexes without voluntary contribution. Further evidence of conditioned pupillary constriction came in the 1930s when both Hudgins (1933) and Baker (1938) reported successful conditioning of pupillary responses using a non-aversive stimulus (a ringing bell) as CS. However, despite several attempts, Steckle & Renshaw failed to replicate Hudgins findings (Steckle & Renshaw, 1934; Steckle, 1936)—leading them to conclude that conditioning of pupillary constriction was not feasible. The same conclusion was reached by Wedell, Taylor & Skolnick (1940), Hilgard, Miller & Ohlson, 1941, Hilgard, Dutton & Helmick, 1949, and Crasilneck & Mccranie (1956), even though both Hilgard and Wedell observed suspected conditioning in a subset of their subjects/experiments.

The question was revisited two decades later when Young (1958) again failed to condition pupillary constriction. Unlike those before him, Young, used a camera to record pupil size. He argued that the methodology employed in previous studies was flawed, leading to improbable measurements (for example CR measured twice as large as UR), and measurements in conflict with normal pupillary behavior, as well as a lack of control of the time between stimulus and response measurement (Young, 1958). Young also designed two additional experiments to investigate the pupillary response in which he used the pupil’s reaction to convergence-accommodation, or an electric shock, as US. None of the experiments revealed any evidence of conditioning. From these results, Young concluded that it is not possible to achieve pupillary conditioning—irrespective of the type of CS used, the duration or intensity of the CS, the ISI, or sex of the subject (Young, 1958).

One decade later Young’s findings were questioned by Voigt (1968), who compared Young’s results with those of Gerall, Sampson & Boslov (1957). In contrast to Young, Gerall et al. reported a possibility to condition pupil dilation using an electric shock as US. Partly in line with the findings reported by Gerall, Sampson & Boslov, 1957 and Gerall & Woodward, 1958, Borrego & Gardner (1986) demonstrated pupillary conditioning using a shock as US. By pairing a CS, in this case consonant-vowel-consonants sounds (CS), with a shock (US), they managed to condition a pupillary response. However, unlike previous studies, Borrego and Gardner used the electrical stimulus to elicit pupillary constriction—not dilation—of the pupil. Later studies have explored additional types of USs. In 2002 Reinhard & Lachnit (2002) reported successful conditioning of the anticipatory pupillary dilation using anticipatory reaction time (serving as US) combined with a non-aversive tone (serving as CS). The importance of the interstimulus interval (ISI) is another matter of contention in the literature. Young (1958) suggests the ISI is not crucial for conditioning while others argue that the ISI is important (Gerall & Woodward, 1958; Fitzgerald & Brackbill, 1968; Borrego & Gardner, 1986). In short, even though there are a handful of studies on pupillary conditioning, there is no consensus regarding what type of US to use, what direction of the CR to look at (dilation or constriction), or what ISI to use. When reviewing all the published literature up until 1993, Loewenfeld & Lowenstein (1999) concluded that conditioning of pupillary responses is not possible and that studies reporting positive results were of poor quality.

Nevertheless, the technology for monitoring eye-movements and pupil size has developed rapidly in the last two decades, and the technological leap from the 1930’s is substantial. Moreover, previous studies have typically used relatively short training protocols, meaning that condition would not have been detected if multiple training sessions is required. Therefore, despite the fact that the evidence overwhelmingly favors the null hypothesis that conditioning of pupillary constriction is not possible, we sought to test this hypothesis again using a modern eye tracker that measures pupil size at a high frequency. This replication attempt was partially motivated by the fact that studying an autonomic response such as the pupillary response rather than a voluntarily controlled response such as the eyeblink response might diminish the problem of volition (see above). Moreover, the pupillary constriction response has a latency of 200–300 ms (Bergamin & Kardon, 2003)—which is substantially faster than the pupil dilation response (Larson et al., 2004), rendering it a suitable object for precise measurements and timing aspects of conditioning.

## Materials & Methods

### Subjects

Eleven participants—six women and five men—aged between 22 and 32 years old—were recruited through convenience sampling to participate in the study. The experimental procedure was explained to the participants orally and in writing and all participants signed an informed consent form at the start of the first testing occasion. For each participant we collected information about age, gender, and degree of visual impairment. The inclusion criteria were: being 18 years of age or older. The exclusion criteria were: severe visual or hearing impairment or disease, at such a degree that would make it difficult to hear the CS or see the US. Participants with milder visual impairment wearing glasses as a visual aid were instructed to go through the test procedure without glasses, as long as they were still able to focus on a dot in the middle of the screen that preceded the US. This study has been reviewed and accepted by the local ethical committee at Lund University, Sweden (dnr: 2017/785).

### Equipment

**Figure 1:**
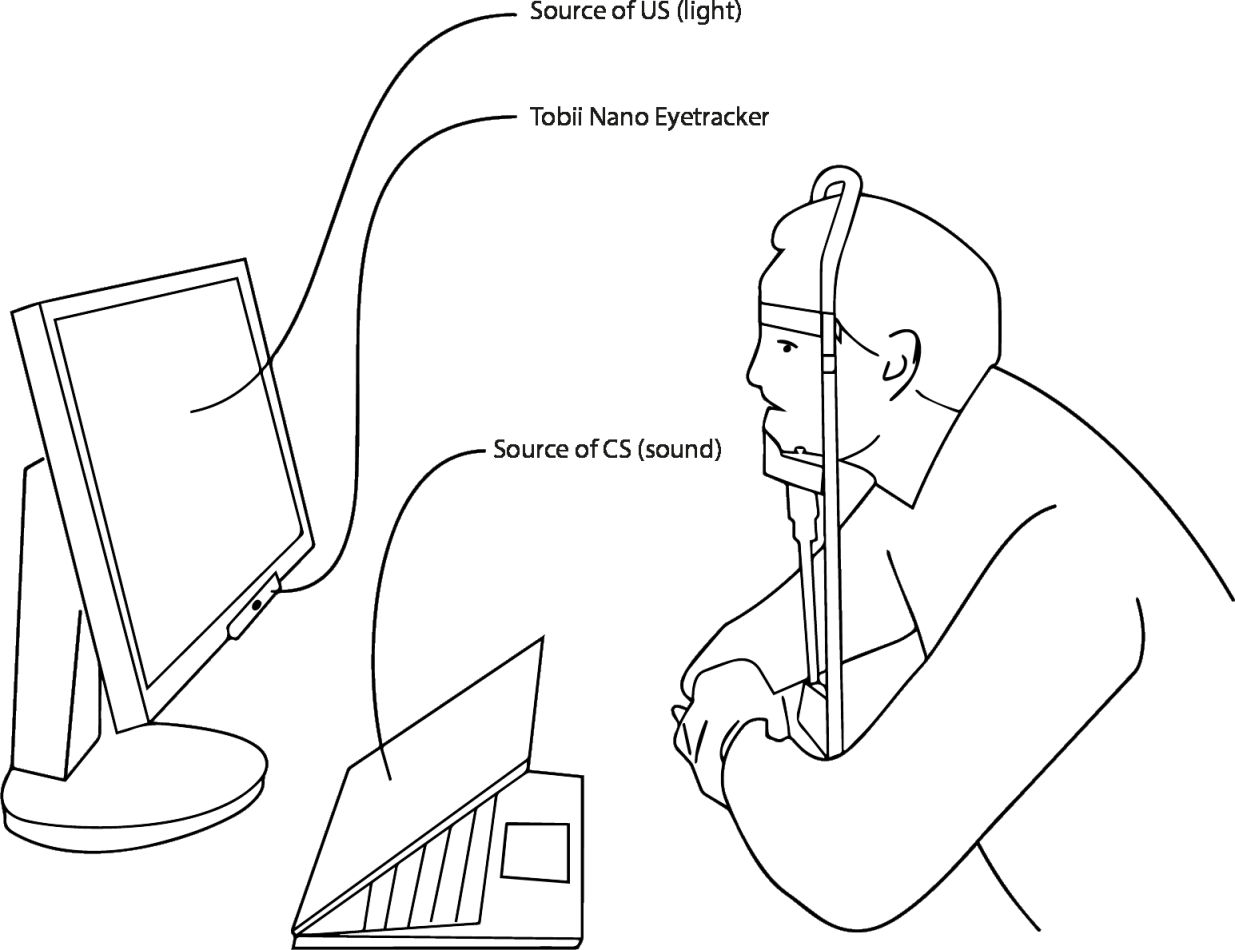
Experimental setup. A participant in a chin rest on a predetermined distance to the computer and the Eyetracker. The sound is played from the computer. The bright light is emitted from the screen.

To maximize the size of the pupils and thus the change in pupil size following the US, the test took place in a darkened room. The hardware consisted of a computer running Windows 10, a 24″screen (Samsung 2443BW), an eyetracker (Tobii Pro Nano), and a chin-rest with an adjustable chin-support to avoid head movements (Fig. 1). Participants were sagittally and horizontally placed in the center of the eyetracker’s headbox and viewed the screen from a distance of 60 cm.

### Procedure

To ensure that a potential lack of conditioning is not due to subjects receiving insufficient training, all participants completed several training sessions. The number of training sessions completed varied from 2–12. Prior to starting the test, each participant was informed about the details of this procedure and asked to try to blink as few times as possible during the trials and also that they should try to blink between trials. To maintain alertness, participants had the option to listen to a podcast or music during the session. This was done through a telephone speaker on low volume to not interfere with hearing the CS clearly. The CS was a 1,000 Hz tone lasting 1 s. The US was a flash of light lasting 500 ms from a computer screen with brightness set to max, sufficient to significantly constrict the pupil. Each session started with two CS alone trials followed by two US alone trials. Thereafter followed 100 trials out of which 25 were probe trials (CS alone) and 75 were paired trials in which CS was followed by US. The sequence of the 100 trials was randomized for each test. Here we used an interstimulus interval of 1,000 ms. Intervals ranging from 500–1,000 ms have been used frequently in eyeblink conditioning tasks and have been shown to yield robust conditioning (McAllister, 1953; Fishbein & LeBlanc, 1967; Wilcox & Ross, 1969; Steinmetz et al., 2011; Kjell, Löwgren & Rasmussen, 2018). Given that the purpose here was to develop an autonomic alternative to eyeblink conditioning we opted to use a similar stimulus configuration.

Trials were separated by 10 ± 2 s. The eyetracker continually measured the pupil size at a frequency of 60 Hz, and throughout each testing session a fixation target was shown (Thaler et al. (2013) ABC in the lower panel of their Fig. 1) that participants were instructed to fixate. As a token of gratitude for contributing to the study, participants received cinema tickets after completed tests.

The auditory and visual stimuli were delivered using custom MATLAB scripts implemented using PsychToolbox (Brainard, 1997; Pelli, 1997) and its PsychPortAudio interface. The eye tracker was controlled, and data recorded, using the Titta toolbox (Niehorster, Andersson & Nyström, 2020).

### Statistical analyses

Timestamps for CS and US onset along with pupil size data from the eyetracker were analyzed offline in Matlab. For each trial, pupil size measurements from the eyetracker from a 5 s time interval, starting at 1 s before CS onset and lasting 3 s after US onset were extracted. We followed the recommendations for processing pupil size data provided by (Mathôt et al., 2018). First, the data was checked for unrealistic pupil size data (pupil sizes smaller than one mm or larger than seven mm), but none were found within the analysis intervals. Second, to remove blink artifacts from the data, five additional samples (83 ms at 60 Hz) were removed from either side of an episode of missing data. Third, to correct for natural variability in pupil size, baseline correction was performed by computing the median pupil size during the 1s interval before CS onset and subtracting this from the entire analysis interval. As such, all pupil sizes reported in this paper are relative to a pre-CS baseline. Finally, pupil size from the left and right eye were averaged. As a measure of conditioning, we examined the minimal size of the pupil on probe trials, *i.e.*, trials that did not have a flash-US, in the interval ranging from 0 to 2 s with respect to CS onset. If conditioning does occur, we would expect the CS to induce a reduction in pupil size following training. Thus, for each training session, we extracted the minimal pupil size on the 25 probe trials and then calculated the average.

To establish the resolution with which pupil size was measured by our setup, we computed the RMS of the change between successive pupil size samples (Niehorster et al., 2020) for each CS alone trial during the entire 5-second analysis window. RMS deviations in pupil size, averaged across trials, revealed that the noise level in the pupil size signal ranged between 0.0070 mm and 0.0128 mm, which is at least an order of magnitude less than any meaningful pupil conditioning effect we may have expected to find, and significantly smaller than attentional effects on pupil size such as reported by Binda & Murray (2015), and Turi, Burr & Binda (2018). As such, the apparatus is sufficiently precise to be able to detect a conditioning effect if it is there.

Data was plotted as histograms and *z*-scores of skewness and kurtosis were calculated to check the distribution of the data. This revealed that data was approximately normally distributed, motivating a parametric test. Thus, to test statistically whether conditioning occurred we used a paired *t*-test to compare the average minimal pupil size on the first and the last session (irrespective of how many sessions the participant went through). To examine any potential correlation between the number of sessions and changes of pupil size, we submitted the minimal pupil size on each trial to a linear mixed model with session number as a fixed effect and participant as a random effect. To further examine the robustness of any null effects we observe, a Bayesian *t*-tests was performed using JASP version 0.16. For this *t*-test, a Cauchy prior with a spread of $1/\sqrt{2}$ was used, which is the default. The strength of the resulting Bayes Factor is interpreted according to the scheme in Van Doorn et al. (2021)

## Results

**Figure 2:**
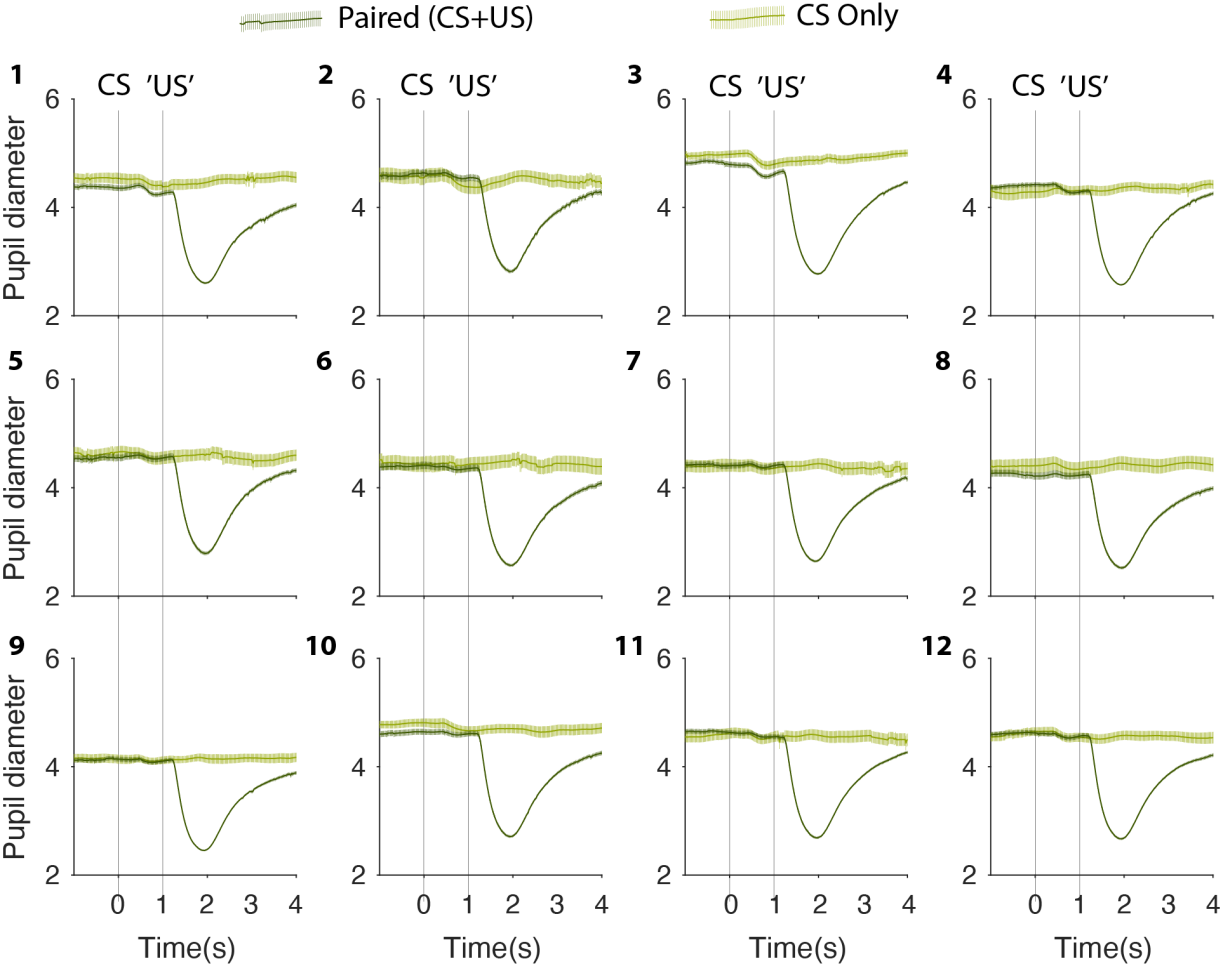
Complete test collection from one participant. Mean pupil size values on paired (CS + US) stimuli (dark green) and CS only (light green) on sessions 1–12. CS, Conditioned Stimulus; US, Unconditioned Stimulus.

Of 11 participants, 10 completed two or more test sessions within a period of 2 weeks. One participant, who due to illness could only complete a single session, was excluded from further analysis. An example of a participant’s complete test results from sessions 1–12 is depicted in Fig. 2. From Fig. 2, it is immediately obvious that whereas trials with a US induce a clear pupillary constriction response of about two mm, there is no comparable change in pupil size on probe (CS-only) trials. This is true on the first test session as well as on the 12th test session.

**Figure 3:**
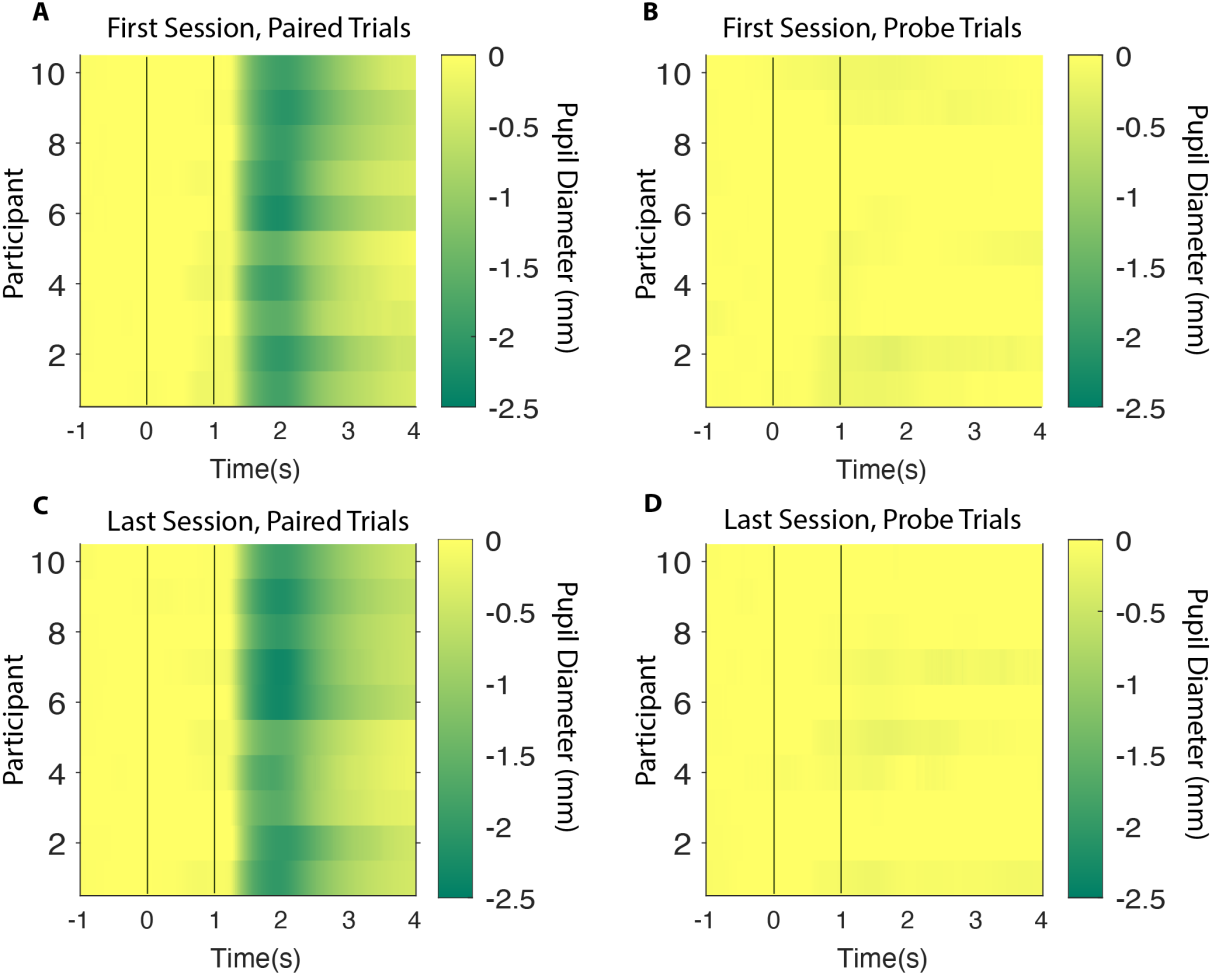
Heatplots. All participants represented as horizontal rows with the colour based on their average pupil size during a time interval of 5 s. CS onset and US onset illustrated as a first and a second vertical white line (A) First session, paired trials (B) First session, probe trials (C) Last session, paired trials (D) Last session, probe trials. CS, Conditioned Stimulus; US, Unconditioned Stimulus.

**Figure 4:**
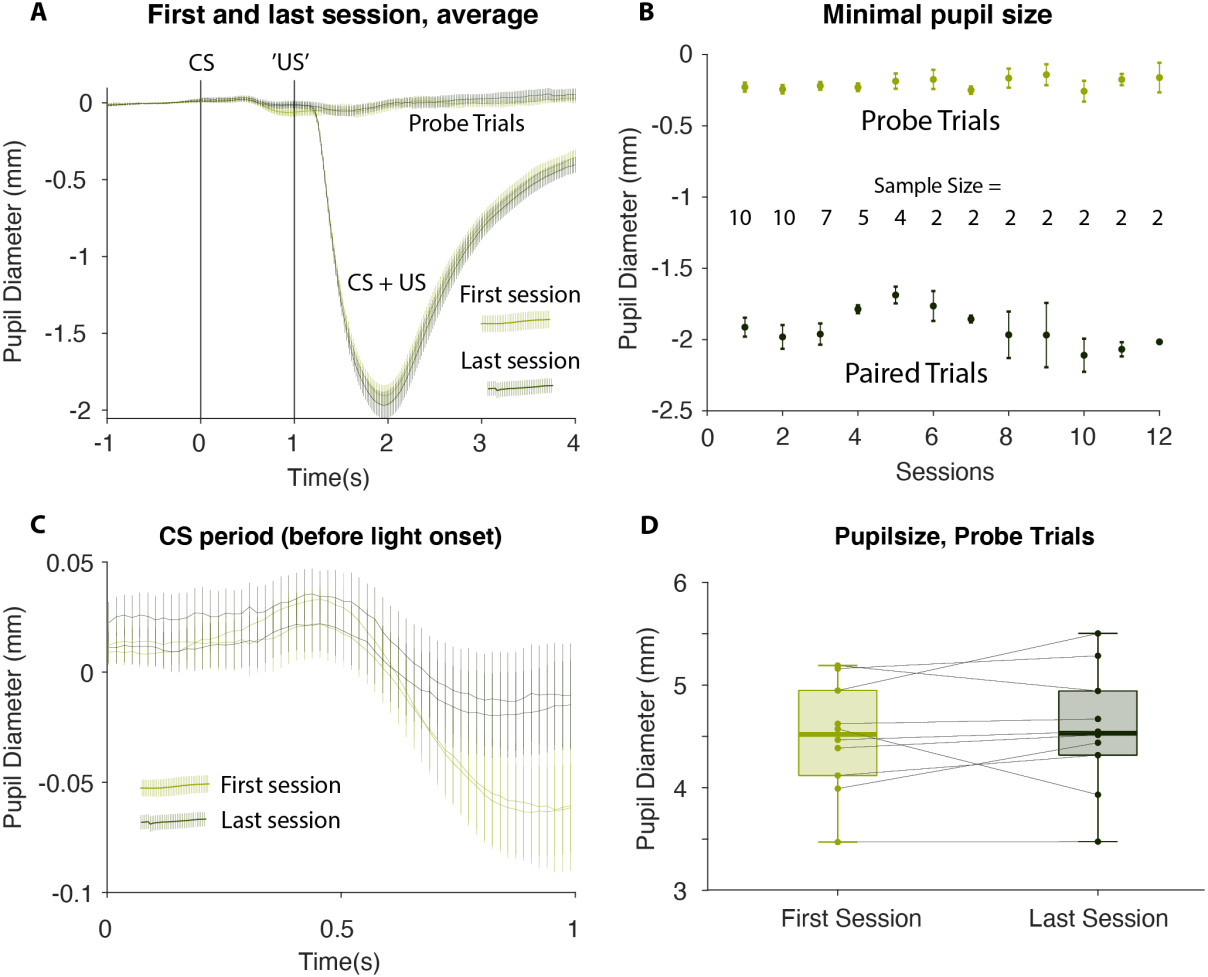
Group analysis. (A) An average of all participants’ first and last test, comparing probe trials to CS+US trials (B) Visualization of the minimal pupil size in the last sessions’ probe and paired trials, as dependent on number of sessions (C) The one second of ISI in first and last session. Left and right pupil visualized (D) All participants’ pupil size in probe trials, and their development from first to last session. CS, Conditioned Stimulus; US, Unconditioned Stimulus; ISI, Interstimulus Interval.

To test this statistically we identified the minimum pupil size on each CS alone trial—irrespective of the exact timing of the minimum but within 2 s from CS onset—and calculated an average for each participant and each session. The results showed that the average minimum pupil size on probe trials was −0.23 ± 0.10 mm (Mean ± SEM) on the first session and −0.19 ± 0.08 mm, on the last session. This 0.043 mm increase was not statistically significant: *t*(9) = 1.01, 95% CI [−0.139–0.053]; *p* = 0.831, suggesting that no conditioning occurred. The corresponding Bayes Factor (BF_+0_ = 0.176) yielded moderate evidence for there being no change in pupil size between the first and the last sessions. Likewise, the mixed linear effect model showed that there was no significant relation between the pupil size on probe trials and the number of test sessions completed (slope 0.005 mm/session ± 0.008 mm/session, *t*(2) = 0.542, *p* = 0.642). In short, there was a complete lack of evidence that conditioning occurred in this experiment (see Figs. 3 and 4).

## Discussion

The aim of this study was to test the hypothesis that the pupillary constriction response to a light stimulus can be conditioned by repeatedly presenting a tone followed by a light. Unlike previous studies this study employed a modern eye tracker that records the size of the pupils precisely and at a high frequency. The results show no evidence of conditioning of the pupillary constriction response. Not even in the two participants who completed 12 conditioning sessions (1,200 trials, of which 900 were conditioning trials), was there any change in pupil size in response to the tone. These findings contradict the findings of Cason (1922) and Baker (1938) who did observe conditioning of pupillary constriction after pairing a sound and a light. However, the results are consistent with the bulk of the literature which shows that conditioning of pupillary responses is not possible (Steckle & Renshaw, 1934; Steckle, 1936; Wedell, Taylor & Skolnick, 1940; Hilgard, Miller & Ohlson, 1941; Hilgard, Dutton & Helmick, 1949; Crasilneck & Mccranie, 1956; Young, 1958; Voigt, 1968).

Even though the study was based on data from only 10 individuals, the protocol included more trials than most previous studies. Since there was not even a hint of conditioning in the data of any of the participants, it seems highly unlikely that adding more participants would change the pattern. Likewise, the fact that the two participants who were trained for 12 sessions showed no conditioning strongly suggests that the amount of training does not affect the outcome. In short, even if conditioning of pupillary constriction occurs when pairing a sound and a light—it would have such an extremely small effect size that it is not feasible as a paradigm to study classical conditioning.

Previous studies have debated the importance of the ISI (Gerall, Sampson & Boslov, 1957; Young, 1958; Gerall & Woodward, 1958; Fitzgerald & Brackbill, 1968). We cannot exclude that a different CS-US interval would yield a different result. However, it is unlikely. All participants verified that they heard the sound clearly, and the US consistently caused a clear constriction of the pupil. In other words, the brain had all the information it needed. Moreover, an interstimulus interval of one second is more than enough time to initiate constriction of the pupil. The US elicited constriction in less than 500 ms (see Figs. 2–4), and since auditory stimuli are processes faster than visual stimuli (Jain et al., 2015), the brain should have sufficient time to respond.

How then can the discrepancy between these results and results from previous studies that successfully conditioned the pupillary response be explained? Could the type of stimuli used and its intensity play a role? There is no consensus in the literature regarding whether such factors are crucial or not (Gerall, Sampson & Boslov, 1957; Young, 1958; Voigt, 1968; Tanck, 1970; Borrego & Gardner, 1986). In this study, the only criterium for the US was that it should elicit a clear constriction of the pupil. A previous study hypothesized that the intensity must be very high when using light as US (Borrego & Gardner, 1986). Perhaps the reason some prior studies managed to condition pupillary constriction, is that the change in pupil size that they observed was part of an emotional response. Young (1958), Lowenstein (1955), and Hess (1965) suggest that emotional excitement influences the pupillary response. The emotional state, as an effect of an electric shock, has even served as US in previous research (Young, 1958). Fitzgerald & Brackbill (1968) suggest that conditioning of the pupillary response might be more dependent on motivational and personality factors, in comparison to somatic responses such as the eyeblink response. Also, Hess (1965) states: “Dilation and constriction of the pupils reflect not only changes in light intensity but also ongoing mental activity” (20, p. 1). It would follow that if the light does not trigger an emotional response, then no conditioning would occur. And, if that is the case—and we believe that it might be –then previous studies have not really examined pupillary conditioning, but rather, conditioning of an autonomic response, of which changes in pupil size is but one component. To test this, future studies would have to record pupil size, as well as another measure related to activation of the autonomous nervous system, such as skin conductance or heart rate.

## Conclusions

This aim of this study was to test whether it was possible to condition pupillary constriction—measured using a precise eye tracker—through repeated pairings of a tone followed by a light. The results show no evidence of conditioned pupillary constriction–not even after 1,200 trials on 12 consecutive training sessions. In conclusion, the data replicate previous findings that pupillary constriction does not show conditioning.

## Supplemental Information

10.7717/peerj.12948/supp-1 Supplemental Information 1 Matlab script

10.7717/peerj.12948/supp-2 Supplemental Information 2 Raw data

## Additional Information and Declarations

### Data Availability

The following information was supplied regarding data availability: The raw data and demographics and one script file that analyses the data are available in the Supplementary Files.
